# Two-dimensional radiographs versus cone-beam computed tomography in planning mini-implant placement: A systematic review

**DOI:** 10.4317/jced.59384

**Published:** 2022-08-01

**Authors:** Gabriela-Franco-da Rosa Caetano, Mariana-Quirino-Silveira Soares, Luciana-Butini Oliveira, José-Luiz-Cintra Junqueira, Monikelly-do Carmo-Chagas Nascimento

**Affiliations:** 1 Division of Oral Radiology, Faculdade São Leopoldo Mandic, Instituto de Pesquisas São Leopoldo Mandic, Campinas, São Paulo (SP), 13045-755, Brazil; 2 Departamento de Cirurgia, Estomatologia, Patologia e Radiologia, Faculdade de Odontologia de Bauru, Universidade de São Paulo, Bauru, São Paulo, Brasil; 3 São Leopoldo Mandic Institute and Research Center, Faculdade São Leopoldo Mandic, Rua José Rocha Junqueira, 13, Campinas, São Paulo, 13045-755, Brazil; 4 Faculdade São Leopoldo Mandic, Instituto de Pesquisas, Division of Oral Radiology, Campinas, Brazil

## Abstract

**Background:**

This study aimed to compare two-dimensional radiographs and cone beam computed tomography (CBCT) images for mini-implant planning.

**Material and Methods:**

A search was performed in PubMed, Embase, Web of Science, Cochrane Library and Google Scholar electronic databases according to PIRD strategy, on September 11, 2021. In vivo studies that compared two-dimensional imaging with CBCT for mini-implant planning were selected. The methodological quality of each study was assessed using the QUADAS-2 tool.

**Results:**

The initial search identified 441 papers. Five studies were added following a manual research. Of the total 446 studies, 40 were selected after title evaluation, 29 remained after abstract evaluation, and 11 were left after full-text analysis. Final screening yielded a total of four studies that composed the narrative synthesis of this systematic review. When comparing the imaging systems for palatal mini-implants, lateral radiographs (LRs) showed approximately the same measurements of bone quantity as those of CBCT, hence bearing no influence on placement site selection. In determining image suitability for interradicular mini-implants, two-dimensional radiographs underestimated the available space.

**Conclusions:**

Lateral radiography is sufficient to quantify the available bone for planning mini-implants installed on the palate, in the median region of upper first premolars. CBCT enhances interradicular mini-implant planning by aiding in implantation site selection, and improving the installation success rate.

** Key words:**Systematic Review, Cone beam computed tomography, Radiography, Orthodontic mini-implant, Dental planning.

## Introduction

Orthodontic mini-implants are important tools for establishing absolute anchorage (1). Clinical studies have shown that mini-implants provide greater predictability (2) and mechanical stability in orthodontic treatment (3). Interradicular sites are used for retraction (4), mesialization (5), distalization (6), intrusion (7), verticalization (8), or traction (9). Paramedian anchorage on the palate is recommended for molar distalization (10), or in cases of maxillary atresia (11). Careful planning for mini-implant placement enables correct anchorage, and averts possible complications, such as injury to anatomical structures (12), root perforations (13), and damage to hard and soft tissues (14). Thus, measures to determine the available bone quantity are essential for selecting the best implantation site (15). The sites for interradicular mini-implant installation are usually evaluated using panoramic and periapical radiographs (16). When the palate is considered a site suitable for implantation, a lateral radiograph (LR) is used for planning (17). However, two-dimensional radiographs have limitations, such as distortion and magnification (15,18).

Cone-beam computed tomography (CBCT) allows a three-dimensional assessment of mineralized tissues in the maxillofacial region, analysis of bone thickness and quality, analysis of root inclination of the adjacent teeth, and identification of anatomical variations (19-23). The planning and selection of sites for osseointegrated implants using CBCT in cases of prosthetic rehabilitation has been shown to reduce complications (24). However, the higher dose of radiation associated with CBCT, compared with two-dimensional radiographic exams, requires careful consideration of its indication in different clinical situations, especially in young patients (25). Several studies (15,19,26,27) have compared two-dimensional radiographs with CBCT for planning mini-implants. Previous systematic reviews have reported the risk of mini-implant failure when these mini-implants come in contact with the root (28), and have evaluated alternative installation sites (18,29,30).

However, to the best of our knowledge, there is no previous systematic review that has assessed how imaging exams can aid in mini-implant planning. Therefore, the main aim of the present systematic review was to evaluate whether the information provided by CBCT and two-dimensional radiographs distinguishes any advantages of one system over the other in planning mini-implants placement.

## Material and Methods

-Protocol and registration

This review was conducted according to the Preferred Reporting Items for Systematic Reviews and Meta-Analyses (PRISMA) (31). The analysis methods and inclusion criteria were specified previously, and registered in the Open Science Framework (OSF) (protocol number 10.17605 / OSF.IO / K5NQX).

-Eligibility criteria

All the studies addressing the following topics were included, according to the PIRD strategy: *in vivo* human population (P) studies; index test (I) of CBCT or CT; reference test (R) for two-dimensional radiographs; diagnosis of interest (D) to determine the amount of bone available for implantation; selection of the site for implantation; and installation success rate.

Randomized and non-randomized clinical trials, as well as cross-sectional and case-control studies conducted on humans, comparing CBCT or CT with two-dimensional radiographs for mini-implant planning, were included. Reviews, letters to the editor, position papers, and case reports or studies that did not compare two-dimensional imaging with CBCT or CT were excluded.

-Search strategy and study selection

Individual searches in the English language were performed in the following databases: PubMed, Embase, Web of Science, and the Cochrane Library. A partial search of the gray literature was conducted using Google Scholar. No time restrictions were applied. All surveys were carried out on September 11, 2021.

The search strategy consisted of a combination of controlled terms (Medical Subject Headings [MeSH] and Emtree terms, respectively) and keywords (“Orthodontics” AND “Mini-implant” AND “Computed Tomography” AND “Dental Radiography”). The reference lists of the included studies and past systematic reviews in the field were also examined manually for additional relevant publications. Duplicates were checked and removed using EndNote Web (Thomson Reuters, Philadelphia, PA, USA).

Two reviewers independently reviewed the titles and abstracts of all the records identified. Subsequently, the full texts of the studies deemed eligible for inclusion were obtained and analyzed. In both the title/abstract and full-text evaluation stages, disagreements were resolved by discussion between the two reviewers. When consensus could not be reached, an experienced third author was consulted.

-Data collection process

The data were extracted independently by two reviewers (MQSS and GFRC) and discussed. The results were updated continuously in an interactive process using the data table. The following data were recorded for qualitative analysis:

• Study characteristics (authors, year of publication, and country) and sample characteristics (type and quantity).

• Characteristics of the intervention (image modality, reference technique, and number of observers).

• Results (type of measures, intra- and interexaminer reliability, clinical applicability), and conclusions.

-Methodological quality assessment

The methodological quality of each study was reviewed critically using the QUADAS-2 tool (Quality Assessment of Diagnostic Accuracy Studies) 32. This tool evaluates four domains: 1) patient selection, 2) index test, 3) reference standard, and 4) flow and time. The clinical applicability of the first three domains was assessed. The study outcomes considered as having good methodological quality were prioritized. Two reviewers made the qualitative assessment of the methodology, and a third author was called upon to discuss and resolve any disagreements, when needed.

The heterogeneity of the studies was analyzed by comparing the extent of participation in the study, methodological points, and appraisal of the results. The studies were separated into two groups to reduce heterogeneity, those focused on palatal mini-implants, and those addressing interradicular mini-implants.

## Results

-Search results

**Figure 1:**
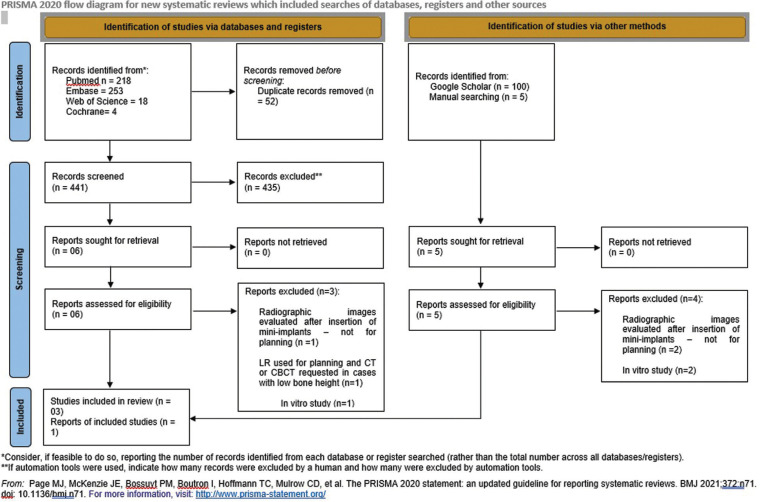
PRISMA Flow Diagram.

The searches conducted in the PubMed, Embase, Web of Science, and Cochrane databases identified 218, 253, 18, and 4 records, respectively, and 100 records were evaluated from the gray literature through Google Scholar. Duplicates were removed manually, resulting in 441 studies. Five additional studies were included by making manual searches and screening reference lists (19,26,33,34,35). The publication dates for these studies ranged from 2002 to 2021. Figure 1 shows the PRISMA flow diagram describing the selection process. After the titles of all the 446 records were screened, 40 articles were deemed eligible for inclusion in the review, and 29 were excluded based on evaluations of the abstracts, leaving 11 studies selected for full-text evaluations. Seven studies (19,26,33,35-38) did not meet the strict inclusion criteria, and were excluded (Appendix 1). Finally, four studies were considered eligible for inclusion in the narrative synthesis of this review (15,27,34,39) because they reported on the CBCT imaging system versus other imaging modalities, or the gold standard clinical techniques for installing mini-implants.

-Study characteristics

Of the four studies included, one evaluated the bone height in the palatal region [15] (Table 1), and three evaluated interradicular mini-implants (27,34,39) (Table 2).

**Table 1:**
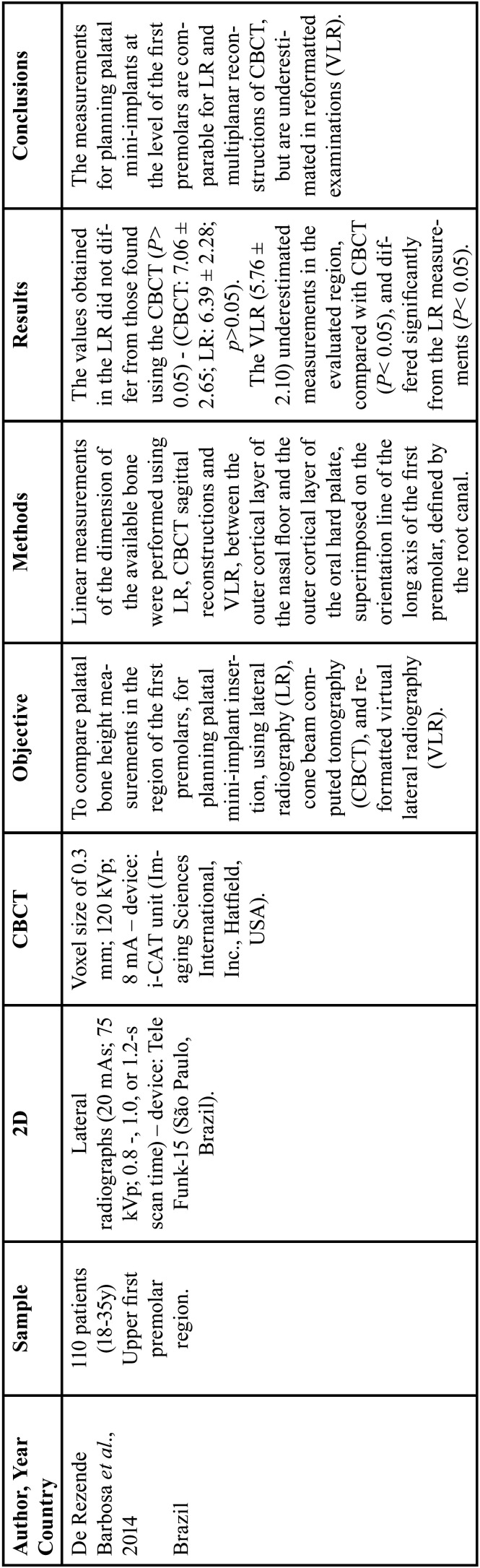
Characteristics of *in vivo* studies that evaluated bone height in the palatal region.

**Table 2:**
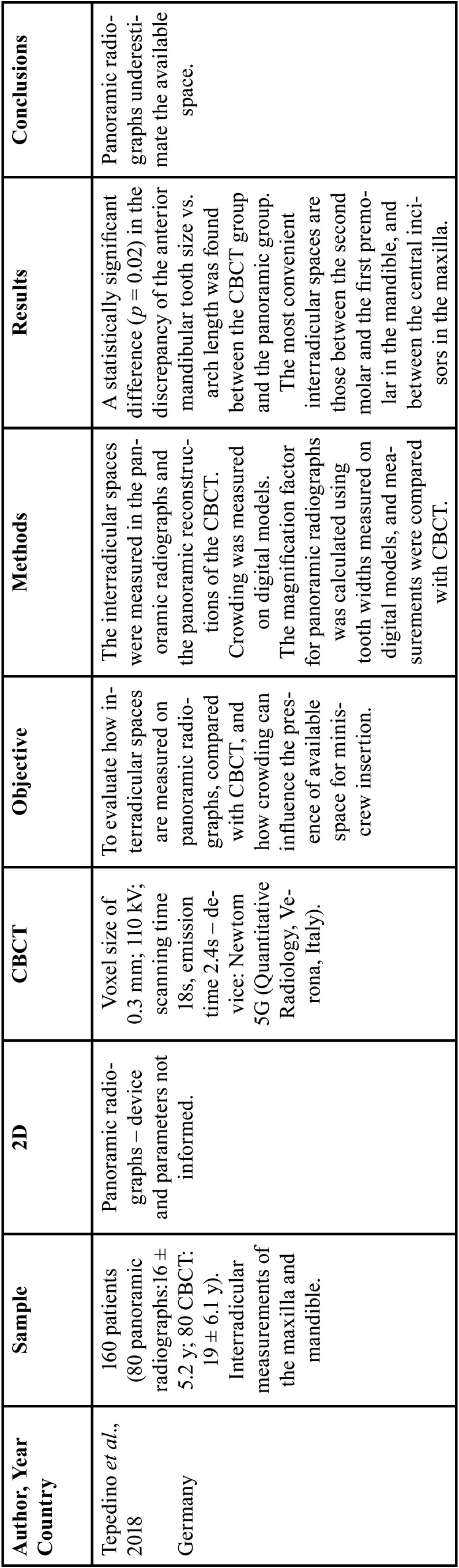
Characteristics of *in vivo* studies that evaluated interradicular mini-implants.

**Table 2 cont.**
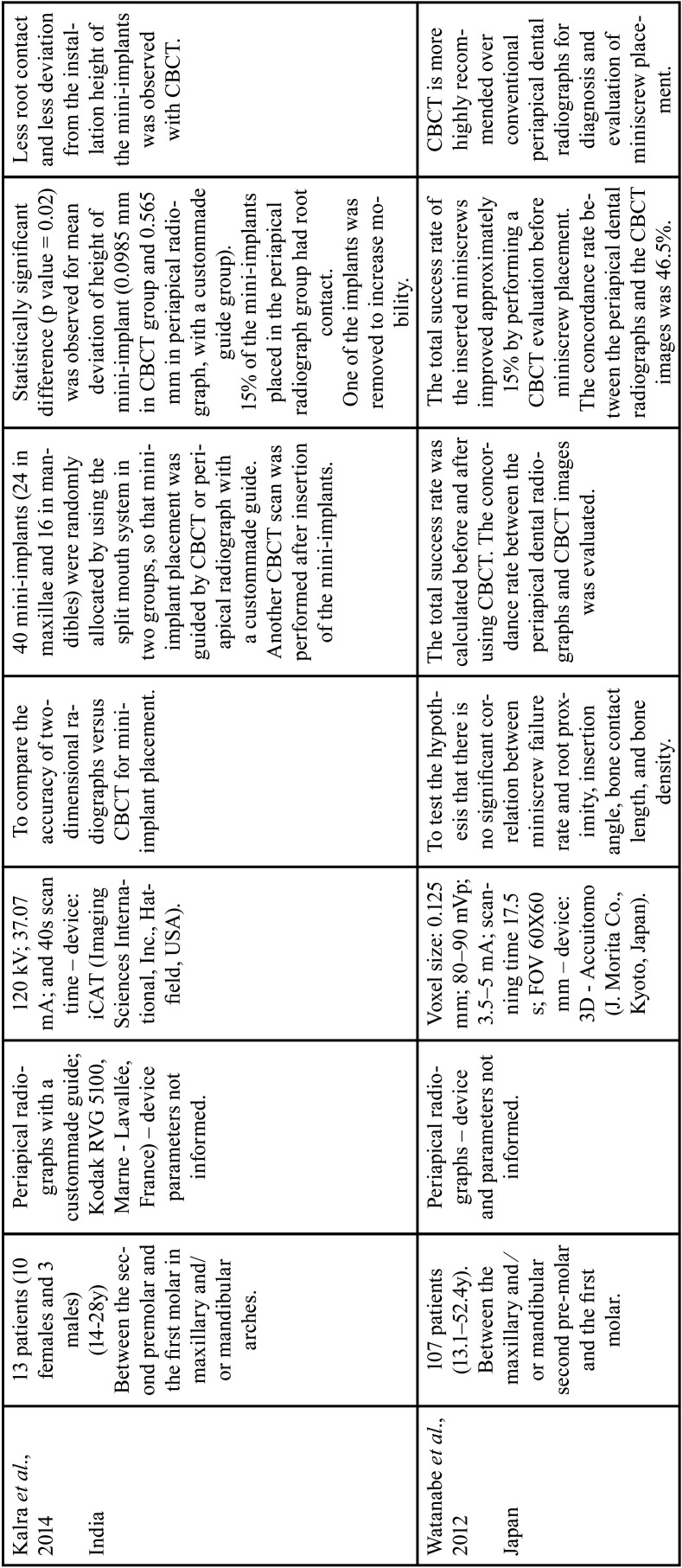
Characteristics of *in vivo* studies that evaluated interradicular mini-implants.

The studies were carried out on patients aged 10-35 years, and provided pertinent inclusion and exclusion criteria. Most of the patients in the studies were female (71.96% in the study by Watanabe *et al*., 2012 (34) 63.12% in the study by Tepedino *et al*., 2018 (27) and 76.92% in the study by Kalra *et al*., 2014 (39). Only one of the studies did not specify this criterion (15).

Two studies used stents (34) or radiographic guides (39) to determine the location and angle of the mini-implant. Paraffin stents and gutta-percha were used during the acquisition of CBCT images (34), and a radiographic guide was used in the periapical radiographs (39).

-Quality assessment of individual studies

The reproducibility of the measurements was assessed heterogeneously among the studies included. One study (27) used only intraobserver agreement, one study (15) used intra- and interobserver agreement (three observers), and two studies (34,39) did not use either of these methods.

De Rezende Barbosa *et al*. (2014) (15) and Tepedino *et al*. (2018) (27) reported no commercial, proprietary, or financial interest in the products or companies described. The other authors did not mention potential conflicts of interest.

-Evaluation of the methodological quality of the studies (Table 3):

**Table 3:**
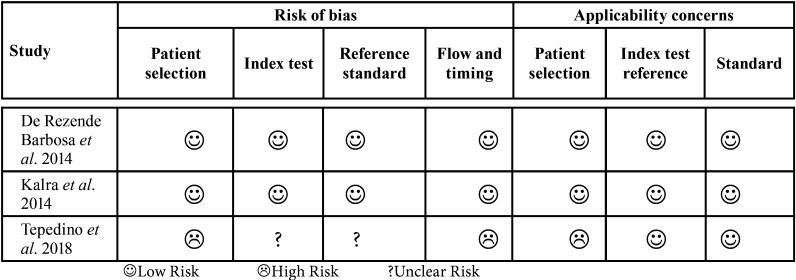
Tabular presentation for QUADAS-2 results of the included studies.

Two studies (15,39) metal the criteria for assessing methodological quality with the QUADAS-2 tool. In one study (27), Domain 1: Patient Selection (Could the selection of patients have introduced bias?) was considered to be at high risk of bias, because it used different patients to compare an examination. All the studies had a low risk of bias due to applicability issues regarding the index test and the reference standard, except for one study (34), which performed the reference test only after placing the mini-implant. In two studies (27,34), the items in Domains 2 and 3 (Could the conduct or interpretation of the index test have introduced bias? / Could the reference standard, its conduct, or its interpretation have introduced bias?) were considered “unclear,” because no information on blinding was reported. On average, the selected studies carried out *in vivo* were considered of good methodological quality according to the QUADAS-2 criteria.

## Discussion

This systematic review assessed whether CBCT is needed for planning mini-implants, whether it contributes to quantifying available bone, and to selecting the implantation site, and whether it improves the installation success rates. Different implantation sites were considered separately when undertaking the analysis.

Although studies assessing the impact of CBCT on mini-palatal implant success could not be identified, one study (15) showed that the measurements taken using CBCT were similar to those obtained using LRs, thus suggesting that CBCT need not be used to estimate the bone available for implantation.

When there are only few anatomical structures on the palate, the amount of available bone becomes one of the main considerations for indicating or contraindicating mini-implant placement (40,41). Thus, the cortical bone height and thickness of the palate at the level of the first and second premolars are more favorable for temporary installation of mini-implants (42), where the distance between the lower cortex of the nasal cavity and the cortical bone of the palate is the greatest (43-46).

The literature shows that measurements for multiplanar reconstructions observed using CBCT versus LRs were very positively correlated, and did not differ significantly (15), in that both indicated the lowest bone height available (35,38). Möhlhenrich *et al*. (2021) (35) recommends using the shortest distance found by the LR for planning mini-implants for the paramedian palatal region, and using the largest distance found by the LR for the insertion of mini-implants in the median palatal region. However, no studies were identified that compared the success of mini-implants performed after planning with the LR versus CBCT, thus suggesting that further investigation in this area is needed.

Tomographic reconstruction simulating LRs underestimated the measurements observed using LRs and CBCT (15). In contrast, the comparison of linear and angular measurements made on conventional two-dimensional cephalometric images for CBCT-generated cephalograms showed the high reproducibility of these measurements, compared with those made on LR images (47-49). Another point regarding CBCT-generated cephalograms is that a larger field of view (FOV) is required, leading to a greater radiation dose absorbed by the body, namely 68 368mSv, compared to approximately 30mSv for digital lateral radiography (50).

The results showed that the benefits provided by CBCT in the planning of interradicular mini-implants lead to a higher installation success rate (34,39), and more accurate assessment of the implant position relative to the adjacent root (27,39). Landin *et al*. (2015) (33) reported similar perforation rates found for planning with two-dimensional methods (60% for periapical radiography, and 50% for panoramic radiography), versus those determined without any radiographic examination (55%). This suggests that two-dimensional imaging examinations do not add any substantial information to the planning process.

In a survey carried out by Tepedino *et al*. (2018) (27), only the regions between the maxillary central incisors, and those from the premolars to the lower second molars showed interradicular distances ≥3 mm. This illustrates the space limitations and difficulties in inserting interradicular mini-implants. Evaluation with two-dimensional radiographs can hinder correct estimation of this space, because of the overlapping of root images, which can also be influenced by the angulation of the X-rays (51,52). In addition, panoramic radiographs are subject to distortions and magnifications that can result in inaccurate measurements (27,53,54).

Two studies (34,39) used guides or radiographic stents. The ideal positioning of orthodontic mini-implants is essential for achieving successful treatment with skeletal anchorage (39). Radiographic guides can provide more accurate locations, (55) and optimize clinical success and treatment safety rates (56). Kalra *et al*. (2014) (39) used a radiographic guide in taking periapical radiographs, designed to assist in the planning of the mini-implants. Those installed with this radiographic examination showed greater height deviation compared with those planned with CBCT, even when associated with the guide. This difference can be attributed to the reference point considered in the exams; that is, the reference point in the CBCT was the orthodontic wire, and that in the periapical radiography was the centralized area between the roots adjacent to the radiographic guide. A higher installation success rate was observed with the mini-implants planned with CBCT.

The studies included met predefined methodological criteria, intended to produce significant results that could be applied in orthodontic practice. The search strategy was designed to include all *in vivo* studies that compared some types of two-dimensional images to CBCT or CT for mini-implant planning. A limited number of studies with heterogeneous methodologies and results were identified. In this review, two protocols were created, one based on the literature addressing studies performing a critical evaluation of diagnostic methods, and a second based on the QUADAS-2 tool for evaluating palatal and interradicular mini-implants (31,32).

The present study had some limitations. The heterogeneity of the included studies limited making any comparisons among them. The variability of the studies and the different mini-implants used precluded predetermining the characteristics, the sample size, the purpose of treatment, the implantation site selection, and the types of two-dimensional examinations, together with their respective radiation doses for image acquisition. Further high-quality primary studies are warranted, considering the clinical relevance of the topic.

In conclusion, lateral radiography is sufficient to quantify the available bone for planning mini-implants installed on the palate, in the median region of upper first premolars. As for interradicular mini-implant planning, CBCT assists in selecting the implantation site, and improves the installation success rate.
